# Health Technology Assessment of Cardiopulmonary Bypass Circuit with and without Phosphorylcholine Coating: A Retrospective Study on Safety and Efficiency in Cardiac Surgery

**DOI:** 10.3390/life14070851

**Published:** 2024-07-06

**Authors:** Ignazio Condello, Giuseppe Nasso, Salvatore Scrivo, Flavio Fiore, Giuseppe Speziale

**Affiliations:** Department of Cardiac Surgery, Anthea Hospital, GVM Care & Research, 70124 Bari, Italy

**Keywords:** cardiopulmonary bypass, phosphorylcholine, cardiac surgery, safety, efficiency, biocompatibility, thrombotic events, coronary artery bypass grafting, mitral valve repair, aortic valve replacement, coating, albumin levels, platelet counts, antithrombin III

## Abstract

Background: Phosphorylcholine has emerged as a potential adjunctive agent in cardiopulmonary bypass (CPB) circuits. Phosphorylcholine serves as a coating for the CPB circuit, potentially enhancing biocompatibility and reducing thrombotic events. However, its impact on specific patient populations and procedural outcomes remains underexplored. Materials and Methods: In this retrospective study, we analyzed data from 60 patients who underwent cardiac surgery with CPB, comprising 20 cases each of coronary artery bypass grafting (CABG), mitral valve repair, and aortic valve replacement. The patient cohort was divided into two groups—30 patients whose CPB circuits were coated with phosphorylcholine (phosphorylcholine-coated group) and 30 patients who did not receive phosphorylcholine supplementation or circuit coating. Both groups underwent surgery with identical CPB circuit designs. We assessed the absence of adverse events, safety, and efficacy parameters, including blood loss, clotting, and the structural integrity of the CPB circuit. Additionally, we measured changes in mean albumin levels (g/dL), mean platelet counts (×10^9^/L), and antithrombin III (ATIII) levels before and after CPB. Results: The retrospective analysis revealed an absence of adverse events in both groups. In the phosphorylcholine-coated group compared to the non-phosphorylcholine-coated group, there was a notable difference in the delta change in mean albumin levels (0.87 ± 0.1 vs. 1.65 ± 0.2 g/dL, *p*-value 0.021), mean platelet counts (42.251 ± 0.121 vs. 54.21 ± 0.194 × 10^9^/L, *p*-value 0.049), and ATIII levels (16.85 ± 0.2 vs. 31.21 ± 0.3 *p*-value 0.017). There was a notable reduction in the perioperative consumption of human complex units after CPB (3 vs. 12, *p*-value 0.019). Conclusions: Both groups, phosphorylcholine and non-phosphorylcholine, demonstrated the absence of adverse events and that the systems are safe for iatrogenic complication. Our findings suggest that the use of phosphorylcholine coating on the CPB circuit, in the absence of supplementary phosphorylcholine, in cardiac surgery is associated with favorable changes in mean albumin levels, mean platelet counts, and ATIII levels. Further research is warranted to elucidate the full extent of phosphorylcholine’s impact on patient outcomes and CPB circuit performance.

## 1. Introduction

Cardiopulmonary bypass (CPB) is a vital component of cardiac surgery, facilitating the circulation of oxygenated blood, while allowing the heart to be temporarily stopped during surgical procedures [1,2]. Despite its essential role, CPB is associated with potential complications, including thrombotic events and systemic inflammation. Phosphorylcholine has recently garnered attention as a potential adjunctive agent in CPB, offering promises of improved biocompatibility and reduced thrombotic risk through its use as a coating for CPB circuits [3,4]. The purpose of this retrospective study is to investigate the safety and efficiency of phosphorylcholine coating circuits on CPB in cardiac surgery. Specifically, we aim to compare outcomes between patients who received phosphorylcholine-coated CPB circuits and those who did not, assessing parameters such as adverse events, blood loss, clotting, and CPB circuit integrity [5,6,7]. By analyzing data from patients undergoing various cardiac procedures, including coronary artery bypass grafting (CABG), mitral valve repair, and aortic valve replacement, we seek to elucidate the impact of phosphorylcholine on specific patient populations and procedural outcomes. Through this comparative analysis, we aim to provide insights into the potential benefits of phosphorylcholine CPB coating circuits in cardiac surgery, while also identifying areas for further research and exploration. Ultimately, a better understanding of phosphorylcholine’s role in enhancing CPB circuit performance and improving patient outcomes could lead to advancements in cardiac surgical practice.

## 2. Materials and Methods

**Figure 1:**
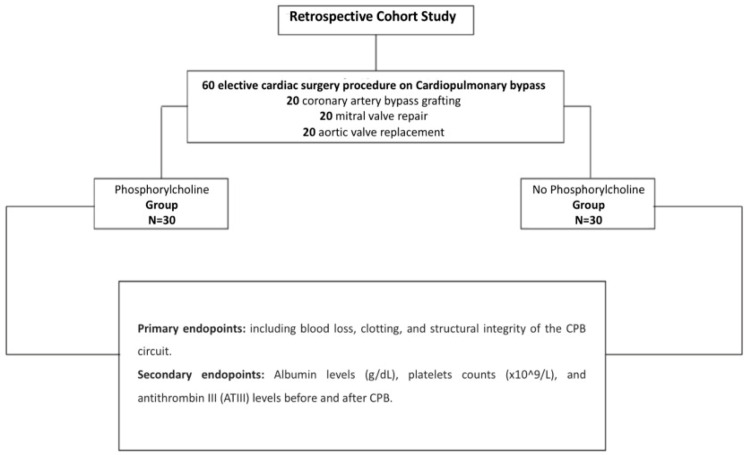
Population and study design.

**Figure 2:**
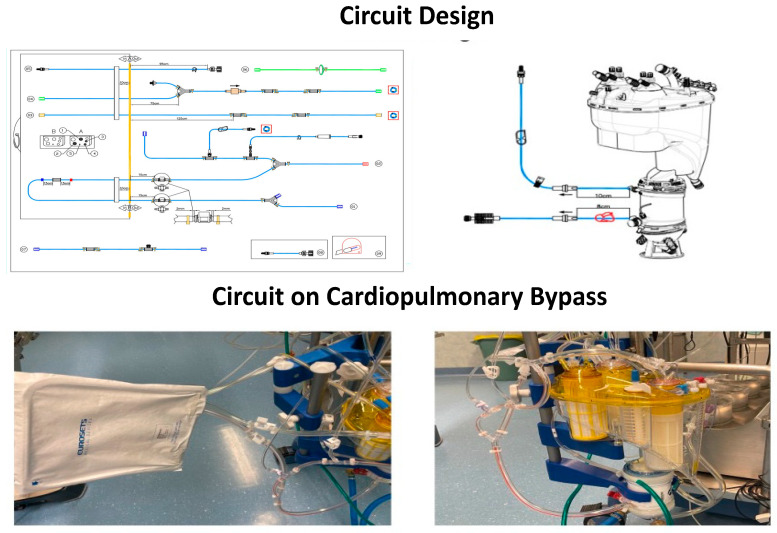
Circuit design and on cardiopulmonary bypass.

This retrospective study analyzed data from 60 patients who underwent cardiac surgery with CPB at Anthea Hospital, GVM Care & Research, Bari, Italy between January and May 2024. The study cohort comprised 20 cases each of coronary artery bypass grafting (CABG), mitral valve repair, and aortic valve replacement. The patient cohort was divided into two groups, as follows: thirty patients whose CPB circuits were coated with phosphorylcholine (phosphorylcholine-coated group) using the A.G.I.L.E. (Advanced Generation Inert Layer ECC) system, based on phosphorylcholine (PC), improving the device’s blood compatibility by reducing platelet adhesion on the coated surface (by Eurosets SRL, Medolla, Italy) and thirty patients who did not receive phosphorylcholine circuit coating (by Eurosets SRL, Medolla, Italy). For the administration of myocardial protection, a closed circuit for blood cardioplegia was used with a heat exchanger (by Eurosets SRL, Medolla, Italy), an infusion syringe pump in sequence, and Saint Thomas solution with procaine; the procedure was repeated every 30 min. Both groups utilized a roller pump, a REMOWELL II venous reservoir, and an A.L.O.N.E AF oxygenator (Eurosets SRL, Medolla, Italy) and followed identical circuit designs (Figure 1 and Figure 2). Patients included in the study met the following criteria: undergoing elective cardiac surgery with CPB, availability of pre- and postoperative laboratory data, and complete medical records. All patients underwent surgery with identical CPB circuit designs. In the phosphorylcholine-coated group, CPB circuits were coated with phosphorylcholine, while the non-phosphorylcholine-coated group received standard CPB circuits without phosphorylcholine supplementation or coating. Preoperative, intraoperative, and postoperative data were collected from electronic medical records. The variables of interest included patient demographics, surgical procedure, CPB duration, adverse events, blood loss, and laboratory parameters. The primary outcomes assessed were the absence of adverse events, including thrombotic events and circuit-related complications. Secondary outcomes included blood loss, clotting, and the structural integrity of the CPB circuit. Additionally, changes in mean albumin levels (g/dL), mean platelet counts (×10^9^/L), and antithrombin III (ATIII) levels before and after CPB were measured. Statistical analysis was performed using appropriate methods based on the distribution of data. Continuous variables were expressed as mean ± standard deviation or median (interquartile range), while categorical variables were presented as frequencies and percentages. Student’s *t*-test or the Mann–Whitney U test was used for comparing continuous variables between groups, depending on the normality of data distribution. Categorical variables were analyzed using the chi-square test or Fisher’s exact test, as appropriate. A *p*-value < 0.05 was considered statistically significant. All statistical analyses were conducted using SPSS 26.0, SPSS Inc., Chicago, IL, USA. This study was conducted in accordance with the principles outlined in the Declaration of Helsinki and was approved by the Internal Institutional Review Board of Anthea Hospital GVM Care & Research, Bari, Italy. Informed consent was waived due to the retrospective nature of the study.

## 3. Results

### 3.1. Patient Characteristics

The study cohort comprised 60 patients, with a mean age of 62 ± 0.4 years (range, 50–67 years). There were 34 (56.6%) male and 26 (43.3%) female patients. The baseline demographic and clinical characteristics were comparable between the phosphorylcholine-coated and non-phosphorylcholine-coated groups (Table 1).

### 3.2. Surgical Procedures and CPB Duration

The distribution of surgical procedures was as follows: in total, 20 (33.3%) patients underwent coronary artery bypass grafting (CABG), 20 (33.3%) underwent mitral valve repair, and 20 (33.3%) underwent aortic valve replacement in each group. The mean duration of cardiopulmonary bypass was similar between the phosphorylcholine-coated and non-phosphorylcholine-coated groups (Table 2).

### 3.3. Adverse Events

There were no adverse events reported in either group, including thrombotic events and circuit-related complications.

### 3.4. Blood Loss, Clotting, and Length of Stay in Intensive Care Unit

The mean blood loss after surgery was 290 ± 75 mL in the phosphorylcholine-coated group and 350 ± 85 mL in the non-phosphorylcholine-coated group. As concerns post-protamine administration, the phosphorylcholine-coated group exhibited a significant decrease in activated clotting time (ACT) (100 ± 12 vs. 135 ± 16 s *p*-value 0.022) compared to the non-phosphorylcholine-coated group. In the phosphorylcholine-coated group compared to the non-phosphorylcholine-coated group, there was a notable reduction in the perioperative consumption of human complex units after CPB (3 vs. 12, *p*-value 0.019); no difference was reported in the length of stay in intensive care unit (ICU) between the two groups (17 ± 2 vs. 18 ± 1, *p*-value 0.71) no adverse events were recorded in the early postoperative course. (Table 3).

### 3.5. Structural Integrity of CPB Circuit

Evaluation of the structural integrity of the CPB circuit revealed no instances of circuit malfunction or damage in either group.

### 3.6. Changes in Laboratory Parameters

A comparison of the preoperative and postoperative laboratory parameters showed differences in mean albumin levels, mean platelet counts, and antithrombin III (ATIII) levels before and after CPB in both groups (Table 4). Notably, the phosphorylcholine-coated group demonstrated a smaller delta change in mean albumin levels (0.87 ± 0.1 vs. 1.65 ± 0.2 g/dL, *p*-value 0.021), mean platelet counts (42.251 ± 0.121 vs. 54.21 ± 0.194 × 10^9^/L, *p*-value 0.049), and ATIII levels (16.85 ± 0.2 vs. 31.21 ± 0.3 *p*-value 0.017) compared to the non-phosphorylcholine-coated group.

## 4. Discussion

Our study findings are in line with the observations reported by De Somer et al. (2000), Schulze et al. (2009), Marguerite et al. (2012) [7], and other cited studies [1,2,3,4]. These studies collectively demonstrate the safety and efficacy of phosphorylcholine-coated CPB circuits, emphasizing the biocompatible nature of phosphorylcholine coating and its ability to maintain platelet function and reduce proinflammatory responses during cardiac surgery [1,2,3,4,5,6,7,8,9], as highlighted by De Somer et al. (2002) and Pappalardo et al. (2006) [10,11,12,13]. Consistent with the findings of De Somer et al. (1999), Marguerite et al. (2012) [7,8], and others, our study confirms the favorable biocompatibility profile of phosphorylcholine-coated CPB circuits [6,7,10,11]. These coatings mitigate platelet activation and preserve platelet count, which may contribute to decreased thrombin formation and improved clinical outcomes, as suggested by Palanzo et al. (1999) [14]. Our study corroborates the observations of De Somer et al. (2002) [10,11,12] and Pappalardo et al. (2006) [13] regarding the potential immunomodulatory effects of phosphorylcholine coating [14]. Smaller changes in laboratory parameters, as observed in our study, further support the hypothesis that phosphorylcholine coating attenuates systemic inflammation and coagulation cascades during CPB, as proposed by Condello et al. (2021) [6]. One notable finding was the absence of adverse events in both the phosphorylcholine-coated and non-phosphorylcholine-coated groups. This suggests that the phosphorylcholine coating does not compromise the safety of CPB circuits and is not associated with an increased risk of thrombotic events or circuit-related complications. These results align with previous studies that have suggested phosphorylcholine’s biocompatible properties, which may contribute to reduced adverse events during CPB [1,2,3,4,5,6,7]. Our study also evaluated efficiency parameters, including blood loss, clotting, and structural integrity of the CPB circuit. We found no significant differences in these outcomes between the phosphorylcholine-coated and non-phosphorylcholine-coated groups, indicating that phosphorylcholine coating does not adversely affect CPB performance. These findings support the hypothesis that phosphorylcholine enhances biocompatibility and reduces thrombotic risk without compromising the functional integrity of the CPB circuit [9,10,11]. Analysis of preoperative and postoperative laboratory parameters revealed interesting trends in the phosphorylcholine-coated group. Specifically, smaller delta changes in mean albumin levels, mean platelet counts, and antithrombin III (ATIII) levels were observed compared to the non-phosphorylcholine-coated group. While the clinical significance of these differences requires further investigation, they may reflect the potential immunomodulatory effects of phosphorylcholine and its ability to mitigate systemic inflammation and coagulation cascades during CPB. In the context of the Health Technology Assessment (HTA), it is important to consider the cost implications of adopting phosphorylcholine-coated circuits [9,10]. The use of phosphorylcholine-coated CPB circuits has the potential to reduce the reliance on human complex units and blood products, which can be costly. By reducing platelet activation and maintaining better hemostatic control, these circuits may decrease the need for additional pharmacological and mechanical interventions to manage bleeding and clotting issues. This reduction in resource utilization could translate into significant cost savings for healthcare institutions, making phosphorylcholine-coated circuits a cost-effective option in cardiac surgery. Several limitations of this study should be acknowledged. Firstly, the retrospective design inherently introduces biases and limits the generalizability of our findings. Additionally, the sample size was relatively small, which may have influenced the statistical power of our analyses. Furthermore, the study was conducted at a single institution, which may limit the external validity of our results. Future research should focus on conducting larger prospective studies with long-term follow-up to further elucidate the impact of phosphorylcholine on patient outcomes with specific inflammatory markers analysis in cardiac surgery. Additionally, mechanistic studies are warranted to better understand the underlying biological mechanisms by which phosphorylcholine exerts its effects on CPB performance and patient physiology.

## 5. Conclusions

In this retrospective study comparing cardiac surgery with cardiopulmonary bypass (CPB) using phosphorylcholine-coated and non-phosphorylcholine-coated circuits, we found that a phosphorylcholine coating was associated with favorable changes in laboratory parameters and comparable safety outcomes. The absence of adverse events, including thrombotic events and circuit-related complications, suggests that phosphorylcholine does not compromise the safety of CPB circuits. Both circuits and systems used in this study are safe; further research is warranted to elucidate the full extent of phosphorylcholine’s impact on patient outcomes and CPB circuit performance. Additionally, efficiency parameters such as blood loss, clotting, and CPB circuit integrity were similar between the two groups, indicating that phosphorylcholine coating does not adversely affect CPB performance. The use of phosphorylcholine-coated CPB circuits has the potential to reduce the reliance on human complex units and blood products, which can be costly. Our findings support the potential benefits of phosphorylcholine in enhancing biocompatibility and reducing thrombotic risk in cardiac surgery. However, further research, including larger prospective studies and mechanistic investigations, is needed to validate these findings and to elucidate the underlying biological mechanisms. Despite the limitations of this study, our results contribute to the growing body of evidence supporting the use of phosphorylcholine as an adjunctive agent in CPB, with the potential to improve patient outcomes in cardiac surgery.

## Figures and Tables

**Table 1:** Patient characteristics. Demographic and clinical characteristics of patients in the phosphorylcholine-coated and non-phosphorylcholine-coated circuits groups.

Characteristic	Phosphorylcholine-Coated Circuits (*n* = 30)	Non-Phosphorylcholine-Coated Circuits (*n* = 30)	*p*-Value
Mean age (y)	62 ± 0.4	63 ± 0.3	0.78
Male sex	18 (60%)	16 (53%)	0.81
Mean body surface area (m^2^)	1.83 ± 0.2	1.86 ± 0.3	0.89
Mean left ventricular ejection fraction (%)	45 ± 5	49 ± 4	0.77
Median NYHA functional class (IQR)	2 (1–3)	2 (1–3)	0.75
EuroSCORE II (mean ± SD)	2.3 ± 0.3	2.5 ± 0.4	0.83
Pre-CPB hematocrit (%)	40.4 ± 1.2	41.03 ± 1.5	0.79
Pre-CPB Hb (g/dL)	13.49 ± 1.1	13.69 ± 1.2	0.80

Note: Values are presented as *n* (%); mean ± standard deviation (SD); or median (interquartile range, IQR) accordingly. NYHA, New York Heart Association; EuroSCORE, European System for Cardiac Operative Risk Evaluation; CPB, cardiopulmonary bypass; Hb, hemoglobin.

**Table 2:** Surgical procedures and CPB duration. Distribution of surgical procedures and mean duration of cardiopulmonary bypass in each group.

Surgical Procedures (*n* = 60)	Phosphorylcholine-Coated Circuits (*n* = 30)	Non-Phosphorylcholine-Coated Circuits (*n* = 30)	*p*-Value
Coronary artery bypass grafting (*n* = 20)	10	10	1.00
Mitral valve repair (*n* = 20)	10	10	1.00
Aortic valve replacement (*n* = 20)	10	10	1.00
CPB time (min.)	97 ± 8	92 ± 4	0.75

Note: Values are presented as *n* or mean ± standard deviation (SD). CPB, cardiopulmonary bypass.

**Table 3:** Blood loss and clotting parameters. Mean blood loss during surgery and clotting parameters in the phosphorylcholine-coated and non-phosphorylcholine-coated groups.

Blood Loss and Clotting Parameters	Phosphorylcholine-Coated Circuits (*n* = 30)	Non-Phosphorylcholine-Coated Circuits (*n* = 30)	*p*-Value
Blood loss after surgery (mL)	290 ± 75	350 ± 85	0.59
Activated clotting time (s)	100 ± 12	135 ± 16	0.022
Human complex (total unit)	3	12	0.019
Length of stay ICU (hours)	17 ± 2	18 ± 2	0.74

Values are presented as *n*; mean ± standard deviation; or as total units.

**Table 4:** Changes in laboratory parameters. Comparison of preoperative and postoperative laboratory parameters, including mean albumin levels, mean platelet counts, and antithrombin III levels in both groups).

Laboratory Parameters	Phosphorylcholine-Coated Circuits (*n* = 30)	Non-Phosphorylcholine-Coated Circuits (*n* = 30)	*p*-Value
***Albumin***			
Preoperative Albumin levels (g/dL)	4.24 ± 0.12	4.58 ± 0.17	
Postoperative Albumin levels (g/dL)	3.37 ± 0.9	2.93 ± 0.2	
Delta Albumin levels (g/dL)	0.87 ± 0.1	1.65 ± 0.2	0.021
***Platelets***			
Preoperative platelet counts (×10^9^/L)	208.95 ± 0.21	212.23 ± 0.19	
Postoperative platelet counts (×10^9^/L)	166.70 ± 0.17	158.02 ± 0.17	
Delta platelet counts (×10^9^/L)	42.25 ± 0.12	54.21 ± 0.19	0.049
**Antithrombin III (ATIII)**			
Preoperative ATII (%)	90.05 ± 0.2	96.04 ± 0.01	
Postoperative ATII (%)	73.2 ± 0.2	64.83 ± 0.02	
Delta ATII (%)	16.85 ± 0.1	31.21 ± 0.12	0.017

Values are presented as *n* (%) or mean ± standard deviation.

## Data Availability

The data are available upon reasonable request from the corresponding authors.
